# Altitudinal Variation in Leaf Nitrogen Concentration on the Eastern Slope of Mount Gongga on the Tibetan Plateau, China

**DOI:** 10.1371/journal.pone.0044628

**Published:** 2012-09-18

**Authors:** Weiqi Shi, Guoan Wang, Wenxuan Han

**Affiliations:** 1 South Subtropical Crops Research Institute, Chinese Academy of Tropical Agricultural Sciences, Zhanjiang, Guangdong Province, China; 2 Department of Environmental Sciences and Engineering, College of Resources and Environmental Sciences, China Agricultural University, Beijing, China; 3 Department of Ecology and Ecological Engineering, College of Resources and Environmental Sciences, China Agricultural University, Beijing, China; Lakehead University, Canada

## Abstract

Mount Gongga spans 6500 m in elevation and has intact and continuous vertical vegetation belts, ranging from subtropical evergreen broad-leaved vegetation to an alpine frigid sparse grass and desert zone. Investigating the altitudinal trends in leaf nitrogen (N) on Mount Gongga can increase our understanding of the global biogeography of foliar N. In this study, 460 leaf samples from mosses, ferns, and seed plants were collected along an altitudinal gradient on the eastern slope of Mount Gongga, and the variation in leaf N concentration (mass basis) with elevation was analyzed. There are considerable differences in leaf N between mosses and ferns, mosses and seed plants, C_4_ and C_3_ plants, and evergreen and deciduous woody plants. The general altitudial pattern of leaf N in Mount Gongga plants was that leaf N kept increasing until an elevation of about 2200 m above sea level, with a corresponding mean annual temperature (MAT) of 8.5°C, and then decreased with increasing elevation. However, the evergreen woody plants displayed a decline trend in leaf N across the altitude gradient. Our findings provide an insight into the altitudinal variation in leaf N.

## Introduction

Nitrogen is generally considered the most limiting element for terrestrial vegetation. The biogeography of leaf nitrogen (N) is a challenging issue and has drawn great attentions [1]–[7]. Reich and Oleksyn found that at the global scale, leaf N concentration (N_mass_) increased from the tropics to mid-latitudes and then remained stable or decreased at high latitudes [3]. Han et al. (2005) showed that in China, leaf N increased with increase in latitude (decreasing mean annual temperature [MAT]) in 753 plant species pooled together [8]. Recently, Han et al. analyzed leaf N in 1900 plant species across China and discovered that leaf N showed remarkable latitudinal and longitudinal trends driven by climate, soil, and plant functional type and that leaf N variation was explained more by precipitation than by temperature [6].

Climate and vegetation-type change markedly with altitude over a short distance, therefore, mountains are ideal sites for examining the biogeographical pattern of leaf N and the effects of climate, soil, and plant species on leaf chemistry. Altitudinal variation in leaf N has been studied intensely [9]–[15], but most of the studies focused on leaf N content per unit leaf area (N_area_) rather than N_mass_. Leaf N_area_ generally tends to increase with increase in altitude, irrespective of life form [9], [10]. However, no conclusive pattern of altitudinal variation in leaf N_mass_ has been detected because relatively few studies have been conducted and a limited number of plant species have been used. For example, Li et al. reported that leaf N_mass_ in the evergreen shrub *Quercus aquifolioides* decreased with increasing altitude [16], whereas Li et al. found that leaf N_mass_ in the deciduous shrub *Hippophae rhamnoides* first decreased and then increased with increasing elevation [15]. Luo et al. observed that leaf N_mass_ in *Picea asperata*, an evergreen conifer tree, decreased below 2950 m and then increased with increasing altitude [14]. Körner reported that leaf N_mass_ increased with altitude in herbaceous plants but was remarkably stable in evergreen woody plants [9]. All these findings were limited by the small number of species used in the studies; therefore, additional studies with a large number of plant species are required to obtain a more general representation of altitudinal variation in leaf N_mass_.

Mount Gongga is an excellent site for examining altitudinal variation in leaf N because of its altitudinal span (from 1100 to 7600 m), abundant species and intact and varied vertical vegetation belts. In this study, we sampled plants from a broad altitudinal range and measured leaf N_mass_. Our objective was to discover the altitudinal variation in leaf N_mass_ of plants in Mount Gongga and to explore the possible causes.

## Materials and Methods

### Study area

Mount Gongga, located on the southeastern side of the Qinghai–Tibet Plateau in Southwest China (29°20′–30°00′N, 101°30′–102°10′E), exhibits remarkable differences in terrain and climate between its eastern and western slopes. The elevation of the eastern slope of Mount Gongga varies from 1100 m (Dadu River valley) to 7600 m. This study was conducted in the Hailuogou region of the eastern slope. Two meteorological observatories are located in the Hailuogou region (at 1640 m and 3000 m). The mean annual precipitation (MAP) and MAT recorded by the two observatories are listed in Table 1. MATs at the sample sites were obtained through linear interpolation on the basis of temperature data recorded. In addition, Zhong et al. used precipitation records from the two observatories, combined with the regional hydrology data, and suggested that rainfall increases with increasing altitude on the eastern slope of Mount Gongga [17].

**Table 1:** Mean annual precipitation (MAP) and mean annual precipitation from 2 meteorological observatories in Hailuogou on the eastern slope of Mount Gongga (Zhong et al. 1997).

Meteorological observatories	Moxi	Sanying
Altitude (m)	1640	3000
MAT(°C)	12.2	4
MAP (mm)	1050	1938

Mount Gongga has an intact and continuous vertical vegetation spectrum that can be observed over a vertical range of 4900 m, from the subtropical arid Dadu River valley to the snowline. A subtropical evergreen broad-leaved vegetation occurs between 1100 and 2200 m with semi-arid shrubs and grasses in the valley below 1500 m. The dominant shrubs and grasses are *Cymbopogon liangshanensis*, *Digitaria sanguinalis*, *Debregeasia longifolia*, *Achnatherum pappiforme*, *Artemisia argyi*, *Buddleja asiatica*, and *Euptelea pleiospermum*; Subtropical evergreen broad-leaved plants grow on the slope with the dominant species being *Cinnamomum camphora*, *Cyclobalanopsis gambleana*, *Morus australis*, *Pyracantha crenulata*; broad-leaved mixed forests distributes from 2200 m to 2800 m with *Cyclobalanopsis gambleana*, *Betula spp.*, *Buddleja asiatica*, *Acer laxiflorum*, *Tetracentron sinense*, *Cinnamomum camphora, Cercidiphyllum japonicum*, and *Euptelea pleiospermum* growing at lower altitudes, with *Tsuga chinensis*, *Acer laxiflorum*, and *Betula spp.* at higher altitudes; frigid dark coniferous forests occurs between 2800 m and 3600 m, and the dominant species are *Tsuga chinensis*, *Picea spp.*, *Abies fabric*; alpine sub-frigid shrub and meadow vegetation is from 3600 m to 4200 m with a variety of *Rhododendron spp.*, *Spiraea spp.*, *Salix spp.*, *Fastuca spp*. and *Carex finitima*; alpine frigid meadow vegetation is between 4200 m and 4600 m with dominant species being *Polygonum viviparum*, *Rhodiola spp.*, *Potentilla fruticosa*, *Kobresia spp.*, *Saxifraga densifoliata* and *Anaphalis lactea*; an alpine frigid sparse grass and desert zone is from 4600 m to 4800 m; an alpine ice and snow zone is above 4900 m.

### Plant sampling

An altitudinal transect from 1200 to 4500 m was established in Hailuogou. Leaves from 291 plant species (total of 460 samples), including 13 mosses, 9 ferns, and 269 seed plants, were collected along the transect at altitudinal intervals of about 100 m in August 2004.

To minimize the influences of human activities, light regime, and location within the canopy, sampling was restricted to open sites with abundant sunshine, far from human habitats. Almost all species found at each sampling altitude were collected. At each site, 5–7 individual plants of each species were sampled, and the same number of leaves was collected from each plant. For all herbs and shrubs, the uppermost leaves of each species were sampled. For tree species, 8 leaves were collected from each individual, and 2 leaves were collected at each of the 4 cardinal directions from the positions of full irradiance, about 8–10 m above the ground. The leaves from the same species of each site were combined into 1 sample.

### Measurement of leaf N_mass_

The plant samples were oven-dried at 65°C and ground to 40 mesh. Leaf N_mass_ was measured using an elemental analyzer (Flash EA1112, CE Instruments, Wigan, UK) in the Stable Isotope Laboratory of the College of Resources and Environmental Sciences, China Agricultural University. The combustion temperature of the elemental analyzer was 1020°C. The standard deviation for the measurement of leaf N_mass_ was 0.1%.

### Ethics statement

No specific permits were required for the described field studies because the location is not privately owned or protected. Our studies did not involve endangered or protected species; thus, no relevant permissions/permits were required for the field studies.

### Data analysis

One-way analysis of variance (ANOVA) and the least significant difference *post hoc* test were used to compare leaf N_mass_ between parallel plant functional groups (mosses versus ferns versus seed plants, herbs versus woody plants, C_4_ versus C_3_ plants, annual versus perennial herbs, and evergreens versus deciduous woody plants).

Linear regression of leaf N_mass_ against latitude was performed to address the altitudinal pattern of leaf N_mass_. Linear regression was also performed for leaf N_mass_ against MAT to show the effect of temperature on variations in leaf N_mass_. Leaf N_mass_ was log10-transformed before regression analyses to improve data normality. The analyses were conducted using the statistical software SPSS 11.0 (SPSS Inc., Chicago, IL, USA).

## Results

### Variations in leaf N_mass_ across all species and between functional groups

**Figure 1:**
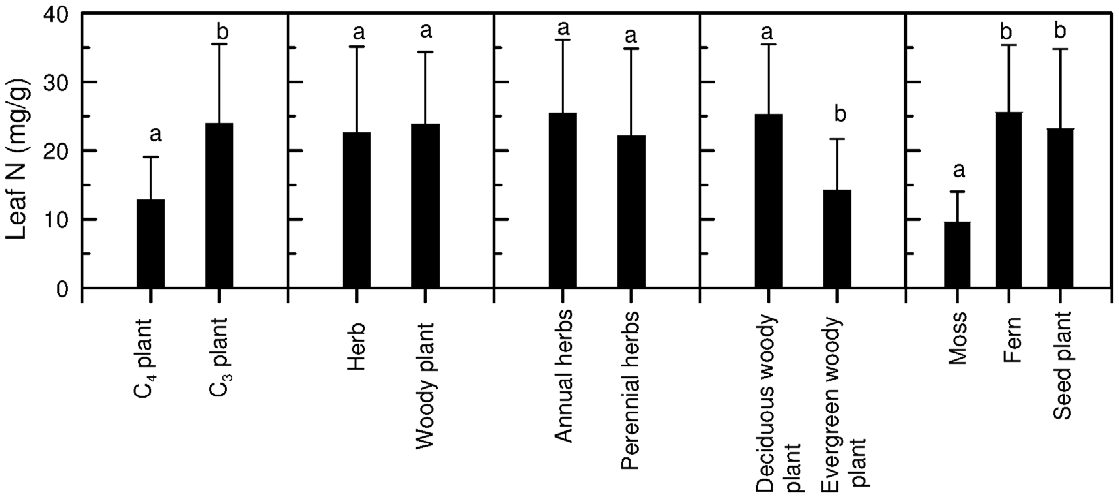
Arithmetic mean values ± SD of leaf N_mass_ across different plant functional groups (different letters indicate significant difference at 0.05 level).

Leaf N_mass_ of Mount Gongga's plants varied greatly, ranging from 2.8 to 59.1 mg/g, across all species. The arithmetic mean N_mass_ ± SD of overall species, seed plants, ferns, and mosses were 22.2±11.9 mg/g (n = 291), 22.5±11.8 mg/g (n = 269), 25.2±10.2 mg/g (n = 9), and 10.0±4.8 mg/g (n = 13), respectively (Fig. 1). One-way ANOVA showed significant differences in leaf N_mass_ between mosses and seed plants and between mosses and ferns (*p* = 0.000).

In seed plants, leaf N_mass_ was significantly higher in C_3_ species (24.2±11.5 mg/g, n = 252) than in C_4_ species (12.8±5.1 mg/g, n = 21) (*p* = 0.000). Leaf N_mass_ was lower in perennial herbs (21.3±12.4 mg/g, n = 119) than in annual herbs (24.0±11.0 mg/g, n = 20), but the difference was not significant (*p* = 0.35). Leaf N_mass_ was slightly higher in woody plants (23.5±10.3 mg/g, n = 136) than in herbs (21.8±12.1 mg/g, n = 139), but the difference between them was not significant (*p* = 0.34). Leaf N_mass_ was remarkably lower in evergreen woody plants (15.6±8.6 mg/g, n = 19) than in deciduous woody plants (24.3±10.1 mg/g, n = 117) (*p* = 0.001).

### Altitudinal pattern of leaf N_mass_

**Figure 2:**
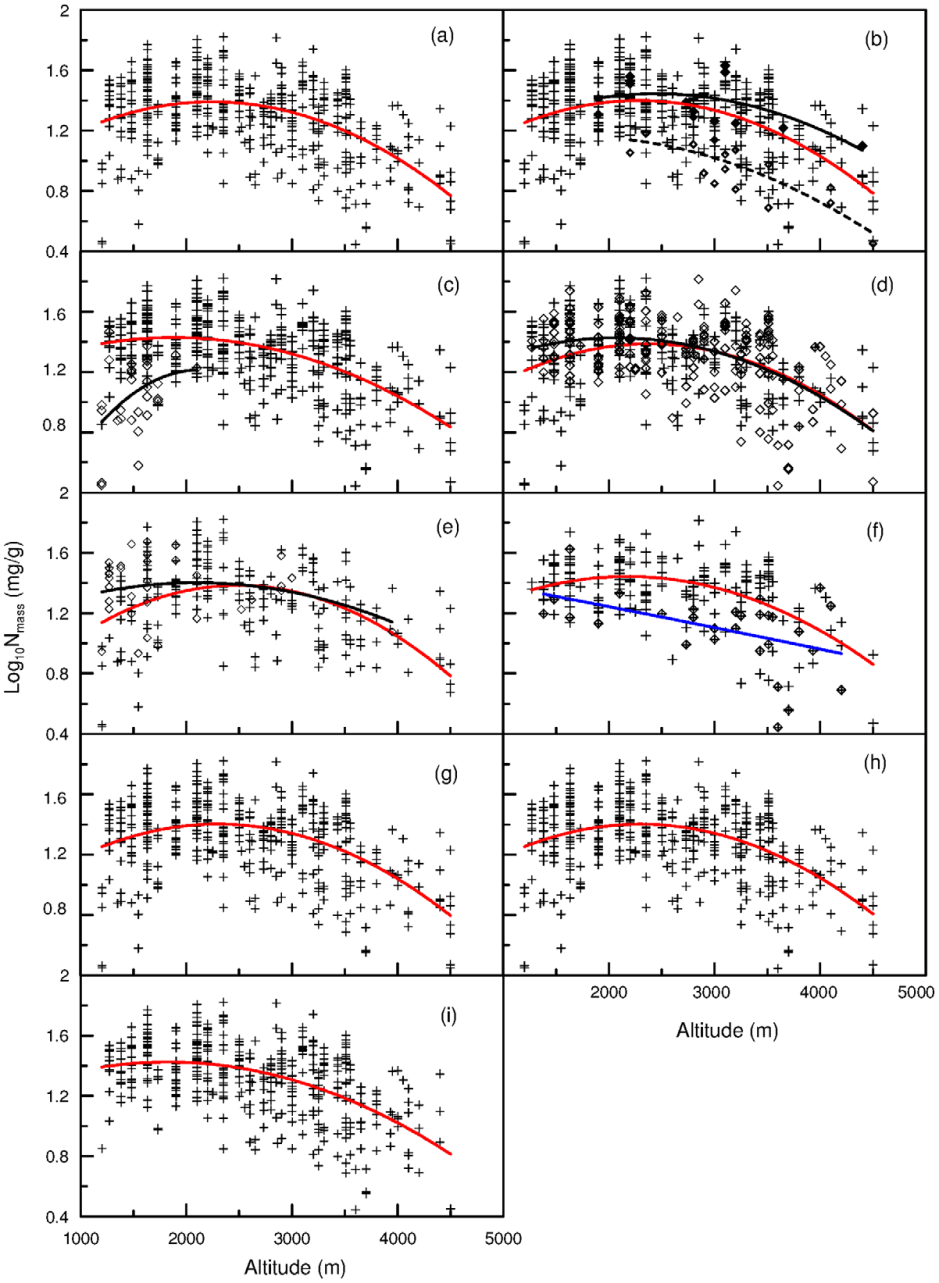
Variation in leaf N_mass_ with altitude. (a) the red line indicates all samples (*R* ^2^ = 0.243, n = 460); (b) those cross dots and the red regression line are seed plants (*R* ^2^ = 0.230, n = 432), those full diamonds and the black solid line represent ferns (*R* ^2^ = 0.397, n = 12), and those empty diamonds and the dash line indicate mosses (*R* ^2^ = 0.651, n = 16); (c) those cross dots and the red line are C_3_ plants (*R* ^2^ = 0.302, n = 408), and those empty diamonds and the dash line indicate C_4_ plants (*R* ^2^ = 0.160, n = 31); (d) the red line and the cross dots are all herbs (*R* ^2^ = 0.213, n = 230), the black line and those diamond are all woody plants (*R* ^2^ = 0.264, n = 214); (e) the red line and those cross dots are perennial herbs (*R* ^2^ = 0.242, n = 196), the black line and those diamonds are annual herbs (*R* ^2^ = 0.045, n = 34); (f) those cross dots and red line indicate deciduous woody plants (*R* ^2^ = 0.242, n = 185), the blue line and those diamond dots with a cross are evergreen woody plants (*R* ^2^ = 0.218, n = 29); (g) the cross dots and the red line are all plants excluding evergreen woody plants (*R* ^2^ = 0.237, n = 431); (h) the cross dots and the red line are all plants excluding mosses (*R* ^2^ = 0.228, n = 444); (i) the cross dots and the red line are all plants excluding C_4_ plants (*R* ^2^ = 0.315, n = 429).

For all species pooled together, leaf N_mass_ showed a significant and nonlinear altitudinal trend, with N_mass_ increasing below about 2200 m and then decreasing with increasing elevation above the altitude (*R* ^2^ = 0.243, *p* = 0.000) (Fig. 2a). Moreover, most plant groups, including seed plants, fern, C_3_ plants, herbs, woody plants, annual herbs, perennial herbs and deciduous woody plants, displayed similar altitudinal trends in leaf N (Fig. 2b–f). In their respective sampling range, leaf N_mass_ in mosses decreased above 2200 m, and leaf N_mass_ increased in C_4_ plants below 2200 m (Fig. 2b, 2c). For evergreen woody plants, leaf N showed a monotonic decline with increasing altitude (Fig. 2f). Fig. 2g, Fig. 2i and Fig. 2j respectively shows that after excluding evergreen woody plants, mosses and C_4_ plants, the remaining plants still had trends similar to the overall altitudianl trend for all species pooled.

### Leaf N_mass_ versus MAT

**Figure 3:**
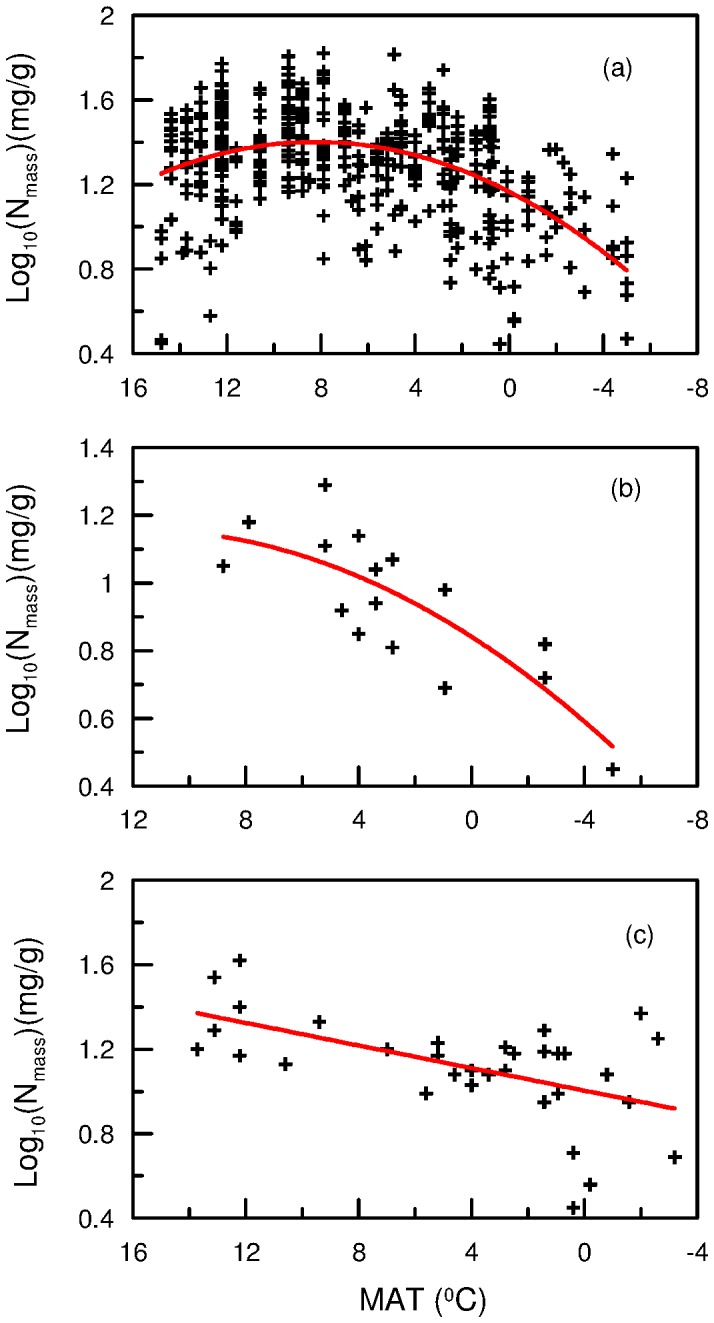
Correlations between leaf N_mass_ and mean annual temperature (MAT). (a) all plants (*R* ^2^ = 0.229, n = 460), (b)moss (*R* ^2^ = 0.651, n = 16) and (c) evergreen woody plant (*R* ^2^ = 0.218, n = 29).

Leaf N_mass_ showed a significant nonlinear relationship with MAT for all plants species pooled together (*p* = 0.000; Fig. 3a). Leaf N_mass_ increased until MAT decreased to about 8.5°C and then decreased with decreasing temperature. Leaf N_mass_ of seed plants, woody plants, herbs, deciduous woody plants, and C_3_ plants all showed an N_mass_–MAT trend similar to that of all plants pooled together (*p* = 0.000). Leaf N_mass_ of C_4_ plants correlated negatively with MAT (*p* = 0.032). For mosses and evergreen woody plants, leaf N_mass_ decreased with a decrease in MAT (*p* = 0.000 and *p* = 0.001, respectively; Fig. 3b, c).

## Discussion

Körner (1989) reported that herbaceous plants displayed an increasing trend in leaf N_mass_ with increasing altitude, but N_mass_ in evergreen woody plants remained stable. Our observations on Mount Gongga are different from those of Körner (1989). For most herbs, we found that leaf N_mass_ first increased and then decreased (Fig. 2e). For evergreen woody plants, we observed a reduction in leaf N_mass_ with altitude. Limited species and samples in the study by Körner (1989) might be responsible for the inconsistency between his findings and ours.

This study shows that about 2200 m elevation was a turning point, which determined the change direction of leaf N with altitude, and the corresponding MAT of the elevation was about 8.5°C (Fig. 3). There are certain comparability between the findings of this study and the observation by Reich and Oleksyn [3]. They found that leaf N decreased with increasing temperature when MAT was greater than 5–10°C but increased with increasing temperature when MAT was lower than 5–10°C.

Leaf N may show opposite responses to temperature: on one hand, leaf N increases as temperature decreases because high leaf N can offset reduced biochemical reaction rates caused by the diminished efficiency of N-rich enzymes at low temperatures (the temperature–plant physiological hypothesis, TPPH); on the other hand, low leaf N may be favored by cold climates because low temperature reduces the rates of decomposition and mineralization of organic matter, resulting in low soil-N availability [3]. Moreover, low temperature may suppress root nutrient uptake (the biogeochemical hypothesis, BH). The altitudinal variation in leaf N may be the result of the competition between the two mechanisms (Fig. 2): except for C_4_ plants, mosses and evergreen woody plants, when MAT was greater than 8.5°C, leaf N of all plants increased with decreasing temperature, suggesting that the influence of TPPH exceeded that of BH; yet, when MAT was lower than 8.5°C, leaf N of all plants declined with decreasing temperature, suggesting that the effect of TPPH was less than that of BH. For the evergreen woody plants, leaf N linearly decreased with increasing altitude (decreasing temperature) (Fig. 2d), and it suggests that in most cases, the influence of BH mechanism on their leaf N may exceed that of TPPH, irrespectively of whether MAT is above or not 8.5°C. For the mosses, because all samples were from the sites above 2200 m (or below 8.5°C), influence of BH mechanism may exceed that of TPPH when MAT was below 8.5°C. How the leaf N of mosses responses to temperature when MAT is above 8.5°C remains unclear. Similarly, for C_4_ plants, we can only suggest that the role of TPPH mechanism may be greater than that of BH when MAT was above 8.5°C, and we do not know the response of C_4_ plants to temperature below 8.5°C.

Precipitation is also an important factor that influences the biogeographic pattern of leaf N_mass_. Han et al. [5] analyzed the leaf N of 1900 plant species across China and suggested that precipitation explained leaf N variation to a greater extent than temperature. On the eastern slope of Mount Gongga, precipitation is expected to increase with elevation [17]. We believe that precipitation played a role in the altitudinal trend in leaf N_mass_ because precipitation increased remarkably with elevation on Mount Gongga. MAP is expected to be greater than 2000 mm when elevation is greater than 3000 m. High soil-water content contributes to the formation of an anaerobic environment, resulting in reduced rates of decomposition and mineralization of organic matter. Consequently, low leaf N_mass_ occurs at higher altitudes. However, we were not able to determine the effect of precipitation because reliable precipitation data were not available.

Species compositional shifts can also affect the overall biogeographic pattern of N_mass_ for all species pooled, because different plants or plant functional types may show significant differences in leaf N (the species composition hypothesis, SCH) [3]. Several comprehensive investigations in China [5]–[8], [18] favored SCH. He et al. [7] suggested that at the biome scale, temperature affects leaf N mainly through a shift in plant species composition rather than by temperature itself. Although large differences in leaf N occurred across functional groups of Mount Gongga's flora, leaf N increased up to an elevation of about 2200 m, and then decreased with increasing elevation, for overall species and for different plant groups except evergreen woody plants, C_4_ plants and mosses (Fig. 2). In fact, only evergreen woody plants displayed an explicit decline trend across the altitude gradient, the altitudinal trend in mosses and C_4_ plants cannot be ascertained. Mosses show similar declining trend of leaf N as the overall species and other plant groups, at least in their distribution range of above 2200 m (Fig. 2b). No mosses were sampled below 2200 m perhaps because the dry and hot climate in the valley does not benefit the growth of mosses. Thus we could not ascertain whether the trend of leaf N in mosses is different from those in other plant groups below 2200 m. For C_4_ plants, leaf N also showed the same trend as other plant groups below 2200 m (Fig. 2c). No C_4_ plants were collected above 2200 m mainly because too low temperature at high altitudes does not favor C_4_ growth. The altitudinal patterns of leaf N in C_4_ plants and mosses were not clear because their samples distributed a limited elevational range. On the eastern slope of Mount Gongga, evergreen woody plants are distributed from 1500 m to 4200 m [19]. Especially at elevations above 2800 m, evergreen woody plants have a high biomass ratio in the community. Still, the number of evergreen species is small, and generally, only 1–3 evergreen woody species exist at each elevation. Thus, evergreen woody plants did not have great contribution to the overall altitudinal pattern of leaf N for all species pooled. Compared with the altitudinal trend in leaf N for all species pooled, after excluding evergreen woody plants, the trend for remaining plants was almost unchanged (Fig. 2g). Similarly, the number of C_4_ plants and mosses also are very small at each altitude; they did not show great influences on the overall altitudinal trend in leaf N because the remaining plants still had the trends similar to all species pooled after excluding C_4_ plants and mosses (Fig. 2i and 2j). The results may suggest that species compositional shifts did not play a major role in the overall altitudinal pattern of leaf N for all species pooled.

In conclusion, the general pattern of leaf N in Mount Gongga's plants was that leaf N first increased up to an altitude of 2200 m, with a corresponding mean annual temperature (MAT) of 8.5°C, and then decreased with increasing elevation. Temperature may be one of influential factors on the altitudinal pattern of leaf N given that the linear relationship between temperature and altitude. Other environmental factors, such as precipitation, air pressure, and herbivores, may also have effects on the altitudinal pattern, but these effects was not explored here for lack of related data. The future studies should address these environmental controls. In addition, more investigations in other mountains are needed to test the altitudinal pattern of leaf N_mass_ revealed here.
